# Tolerability of NGX-4010, a capsaicin 8% dermal patch, following pretreatment with lidocaine 2.5%/prilocaine 2.5% cream in patients with post-herpetic neuralgia

**DOI:** 10.1186/1471-2253-11-25

**Published:** 2011-12-19

**Authors:** Lynn R Webster, Margarita Nunez, Marvin D Tark, Edwin D Dunteman, Biao Lu, Jeffrey K Tobias, Geertrui F Vanhove

**Affiliations:** 1 Lifetree Clinical Research, Salt Lake City, UT, USA; 2 Comprehensive NeuroScience, Inc., St. Petersburg, FL, USA; 3 Drug Studies America, Marietta, GA, USA; 4 A & A Pain Institute of St. Louis, St. Louis, MO, USA; 5 NeurogesX, Inc., San Mateo, CA, USA

## Abstract

**Background:**

Post-herpetic neuralgia (PHN) is a common type of neuropathic pain that can severely affect quality of life. NGX-4010, a capsaicin 8% dermal patch, is a localized treatment that can provide patients with significant pain relief for up to 3 months following a single 60-minute application. The NGX-4010 application can be associated with application-site pain and in previous clinical trials pretreatment with a topical 4% lidocaine anesthetic was used to enhance tolerability. The aim of the current investigation was to evaluate tolerability of NGX-4010 after pretreatment with lidocaine 2.5%/prilocaine 2.5% anesthetic cream.

**Methods:**

Twenty-four patients with PHN were pretreated with lidocaine 2.5%/prilocaine 2.5% cream for 60 minutes before receiving a single 60-minute application of NGX-4010. Tolerability was assessed by measuring patch application duration, the proportion of patients completing over 90% of the intended treatment duration, application site-related pain using the Numeric Pain Rating Scale (NPRS), and analgesic medication use to relieve such pain. Safety was assessed by monitoring adverse events (AEs) and dermal irritation using dermal assessment scores.

**Results:**

The mean treatment duration of NGX-4010 was 60.2 minutes and all patients completed over 90% of the intended patch application duration. Pain during application was transient. A maximum mean change in NPRS score of +3.0 was observed at 55 minutes post-patch application; pain scores gradually declined to near pre-anesthetic levels (+0.71) within 85 minutes of patch removal. Half of the patients received analgesic medication on the day of treatment; by Day 7, no patients required medication. The most common AEs were application site-related pain, erythema, edema, and pruritus. All patients experienced mild dermal irritation 5 minutes after patch removal, which subsequently decreased; at Day 7, no irritation was evident. The maximum recorded dermal assessment score was 2.

**Conclusion:**

NGX-4010 was well tolerated following pretreatment with lidocaine 2.5%/prilocaine 2.5% cream in patients with PHN. The tolerability of the patch application appeared comparable with that seen in other studies that used 4% lidocaine cream as the pretreatment anesthetic. This study is registered at http://www.clinicaltrials.gov as number NCT00916942.

## Background

Neuropathic pain is pain arising as a direct consequence of a lesion or disease affecting the somatosensory system [1]. Post-herpetic neuralgia (PHN) is a common type of neuropathic pain occurring as a complication of reactivation of the varicella zoster virus (shingles). PHN is caused by damage to the small-diameter sensory C and Aδ fibers within primary afferent neurons, which results in hypersensitivity and exaggerated responses to normally innocuous stimuli (evoked pain), as well as both continuous and paroxysmal spontaneous pain [2-6]. Symptoms of PHN include pain from normally non-noxious stimuli such as the brush of clothing (allodynia), increased sensitivity to painful stimuli (hyperalgesia), intermittent stabbing or lancinating pain and constant deep burning [2,7]. The pain caused by PHN can be highly debilitating and can severely affect patients' quality of life [2,5,6].

Neuropathic pain therapies include adjunctive analgesic antidepressants and anticonvulsants, as well as opioids and various topical treatments such as lidocaine or low-concentration capsaicin [8,9]. These treatment options commonly provide pain relief in only a subset of patients with neuropathic pain, and the relief that is achieved is often only partial [6]. Additionally, systemic treatments can be accompanied by a significant side effect burden that can include dizziness, somnolence, and nausea. There are also a number of contraindications and potential drug-drug interactions to take into account with systemic neuropathic pain treatments, especially in the often elderly population of patients with PHN [6,9]. Together, these factors highlight the unmet need in the treatment of PHN.

A recent development for the treatment of patients with PHN is a capsaicin 8% w/w dermal patch (QUTENZA™, NGX-4010). Capsaicin is a selective agonist of Transient Receptor Potential Vanilloid 1 (TRPV1) receptors, which are activated by noxious heat, low pH, and some endogenous inflammatory mediators [10,11]. TRPV1 receptors are ligand-gated cation channels and are expressed on the majority of C and Aδ fibers [10,12]. Exposure of TRPV1 receptors to high concentrations of capsaicin initially causes depolarization, action potential initiation, and burning pain. However, this is followed by a reversible defunctionalization and reduction of epidermal nerve fibers [12,13] resulting in a prolonged inhibition of pain transmission. NGX-4010 has been demonstrated to provide significant pain relief for up to 3 months to patients with PHN or HIV-associated neuropathy [12,14-17].

Administration of NGX-4010 is associated with pain, erythema, and other application-site reactions. In previous clinical trials, 4% lidocaine cream was used as pretreatment and the procedure appeared well tolerated, with the great majority of patients receiving treatment for over 90% of the intended treatment duration [12,14-17]. The objective of the current study was to evaluate the tolerability of NGX-4010 after pretreatment with an alternative topical anesthetic, lidocaine 2.5%/prilocaine 2.5% anesthetic cream, in patients with PHN, since 4% lidocaine cream is not available in all of the countries in which NGX-4010 is approved. Lidocaine 2.5%/prilocaine 2.5% cream is a eutectic mixture of local anesthetics (EMLA™) and a commonly available topical anesthetic formulation. The efficacy of lidocaine 2.5%/prilocaine 2.5% cream has been shown to be comparable with that of 4% lidocaine alone in a number of procedures, such as prior to chemical peeling, minor surgical procedures, or laser hair removal [18-20].

## Methods

### Patients

Patients aged 18-90 years with pain due to PHN that persisted for at least 3 months after shingles vesicle crusting and was of appropriate severity for treatment with NGX-4010 in the opinion of the investigator, were eligible for study entry. Patients were not permitted to take part if they were already receiving opioid medications, unless these were taken orally or transdermally and the daily dose did not exceed 60 mg/day of morphine or equivalent. Patients were not permitted to use any topical pain medications on the affected areas within the 7 days preceding treatment or during the study. Female patients of childbearing age were required to have had a negative pregnancy test at the screening visit, and be using an effective method of contraception throughout the study period. The study was conducted in accordance with the Declaration of Helsinki, was consistent with Good Clinical Practice guidelines, and was approved by the following institutional review board: Aspire IRB, LLC, 9320 Fuerte Drive, La Mesa, CA. Written informed consent was obtained from all participating patients before the study commenced.

### Procedures

This was a 7-day, open-label, non-controlled, non-randomized, multicenter study with a total of three visits--Screening, Day 0 (treatment day), and Day 7. All patients were assigned to receive open-label NGX-4010 (QUTENZA™, NeurogesX Inc., San Mateo, CA, USA), a capsaicin 8% w/w (640 μg/cm^2^) patch. Patients could receive up to four patches (280 cm^2 ^each) corresponding to a total treatment area of 1,120 cm^2^. Painful areas were identified and pretreated with a thick layer of lidocaine 2.5%/prilocaine 2.5% cream (1-2 g/10 cm^2^), then covered with Tegaderm™ Film (3M Health Care, Neuss, Germany) for 60 minutes to prevent drying out. After the anesthetic cream was removed, the skin was washed with soap and water, and dried. NGX-4010 patches were cut to size and applied to the pretreated areas for 60 minutes. Patients were permitted to use immediate-release, opioid-based, oral pain medications such as oxycodone hydrochloride oral solution (1 mg/mL concentration) in the clinic as needed for relief of treatment-related discomfort. Local cooling measures could also be used following patch removal. Patients were permitted to take hydrocodone bitartrate/acetaminophen (5/500 mg) as needed for up to 5 days following treatment.

### Assessments

This was primarily a safety study so there were no assessments for the efficacy of NGX-4010. Safety and tolerability analyses were performed on all enrolled patients who received study drug.

To determine the tolerability of NGX-4010 following pretreatment with the lidocaine 2.5%/prilocaine 2.5% anesthetic cream, the following were assessed: mean duration of patch application; mean changes in "Pain Now" NPRS scores from pretreatment values on the day of treatment; the proportion of patients using analgesic medication for treatment-associated pain during and following patch application on the day of treatment and on Days 0 to 7 and the mean daily dose; and the proportion of patients completing at least 90% of the intended patch application duration. The sample size for this study was based on clinical judgment to adequately assess the tolerability of NGX-4010 in conjunction with a topical anesthetic formulation for the treatment of PHN.

Adverse events (AEs) were monitored throughout the study from application of the topical anesthetic to study end. AEs were mapped to preferred term and system organ class using the Medical Dictionary for Drug Regulatory Activities (MedDRA, version 10.1). Vital signs, including blood pressure and heart and respiratory rates, were monitored at all study visits. On the treatment day, vital signs and NPRS "Pain Now" scores were recorded immediately prior to anesthetic application, 30 and 55 minutes after anesthetic application, 25 and 55 minutes after patch application, and 5, 25, 55, and 85 minutes after patch removal. In addition, "Pain Now" NPRS scores were recorded on the evening of the treatment day.

Dermal assessments were carried out at all study visits and on the treatment day, before anesthetic application, within 5 minutes of its removal, and following patch removal within 5 minutes and at 25, 55, and 85 minutes, using the following scoring system [21]: 0 = no evidence of irritation, 1 = minimal erythema, barely perceptible, 2 = definite erythema, readily visible; minimal edema or minimal papular response, 3 = erythema and papules, 4 = definite edema, 5 = erythema, edema, and papules, 6 = vesicular eruption, 7 = strong reaction spreading beyond test site.

## Results

### Patient disposition

A total of 25 subjects were enrolled in the study and 24 received study drug treatment. One patient did not receive treatment with NGX-4010 due to an increase in blood pressure that occurred during treatment with the topical anesthetic. Three patients (12%), including the patient who did not receive NGX-4010 treatment, withdrew from the study prematurely; the other two withdrawals were due to scheduling conflicts.

### Patient demographics

The average age of patients enrolled in the study was approximately 67 years (Table 1). The mean duration of PHN pain was 5.5 years and the mean baseline NPRS score was 5.5. One-third of the patients (eight in total) had previously received NGX-4010 treatment.

**Table 1:** Patient Demographics

	NGX-4010 (n = 24)
Age (years)	
Mean (SD)	66.9 (11.8)
Min, max	39, 83
Gender, n (%)	
Male	13 (54)
Race, n (%)	
White	23 (96)
African American	1 (4)
Other	0
Duration of PHN pain (years)	
Mean (SD)	5.5 (4.3)
Min, max	1.0, 19.3
Pain level at screening	
Mean (SD)	4.3 (2.2)
Min, max	0, 9.0
Baseline pain level	
Mean (SD)	5.5 (1.7)
Min, max	2.1, 8.9

Abbreviations: PHN; post-herpetic neuralgia, SD; standard deviation.

### Safety and tolerability

#### Treatment

The intended duration of treatment was 60 minutes; the observed mean duration of NGX-4010 patch application was 60.2 minutes (Table 2). Four patients received NGX-4010 treatment for longer than 60 minutes and 1 patient had the patch removed after 59 minutes. All 24 patients completed at least 90% of the intended patch application duration. The mean application duration of the topical anesthetic was 60.9 minutes and the mean surface area treated with NGX-4010 was 407 cm^2^.

**Table 2:** Extent of Exposure to Lidocaine 2.5%/Prilocaine 2.5% and NGX-4010

	NGX-4010 (n = 24)
Duration of topical anesthetic application (minutes)	
Mean (SD)	60.9 (3.0)
Min, max	56, 72
Duration of NGX-4010 patch application (minutes)	
Mean (SD)	60.2 (0.7)
Min, max	59, 63
Surface area treated (cm^2^)	
Mean (SD)	407 (301)
Min, max	59, 1000
Number of subjects completing at least 90% of intended NGX-4010 patch application time, n (%)	24 (100)

Abbreviations: SD; standard deviation.

#### Pain Associated with Treatment

**Figure 1:**
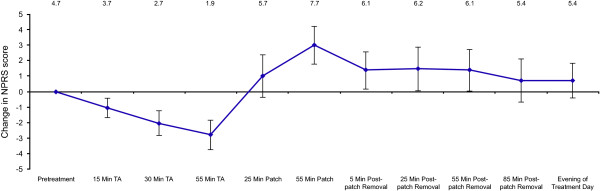
**Change in pain on the treatment day**. Patients recorded their "Pain Now" NPRS scores on the day of treatment before and during application of lidocaine 2.5%/prilocaine 2.5% cream, during NGX-4010 administration, up to 85 minutes following patch removal, and on the evening of treatment day. The results represent the change in mean (± 95% CI) "Pain Now" NPRS scores from the mean pretreatment score at each time point. The mean absolute NPRS scores are also provided. CI; confidence interval, NPRS; Numeric Pain Rating Scale, TA; topical anesthetic.

The mean absolute NPRS score was 4.7 before treatment, decreased following anesthetic application and then increased following application of NGX-4010 (Figure 1). A maximum mean increase in NPRS score of 3.0 was recorded just before removal of NGX-4010. The score quickly declined following patch removal to less than half the maximum within 5 minutes of patch removal (+1.4) and returned to near pre-anesthetic levels (+0.7) within 85 minutes of patch removal (Figure 1). Four patients (17%) had no increase in pain from the pretreatment level during patch application or after removal. On the evening of the treatment day, mean NPRS scores remained at near pretreatment levels (Figure 1).

Half of the patients treated with NGX-4010 received medication on the day of application (study Day 0) for treatment-related discomfort. A total of 10 patients (42%) received immediate-release, opioid-based analgesic medication (oxycodone or oxycodone-based medication), 3 patients (13%) received hydrocodone (or hydrocodone-based medication), and 1 patient (4%) received acetaminophen. The mean dose of opioid medication received on Day 0 was 11.5 mg for oxycodone and 15.0 mg for hydrocodone. The number of patients receiving medication for treatment-related discomfort decreased rapidly after the day of NGX-4010 treatment, with only 4 patients receiving medication on Day 1, 2 patients on Day 3, 1 patient on Days 5 and 6 and no patients on Day 7.

#### Adverse Events

All 24 patients within the study reported at least one treatment-emergent AE (Table 3). The most common treatment-emergent AEs were application-site related, with 100% of patients reporting application-site erythema, 92% reporting application-site pain, 17% reporting application-site edema, and 4% reporting application-site pruritus (Table 3). There was one case of increased blood pressure considered to be treatment related; however, this was mild and resolved on the day of treatment. The majority of patients reported treatment-emergent AEs that were mild to moderate in severity. Application-site pain was the only severe treatment-emergent AE and was reported by 3 patients (13%; Table 3). All application-site AEs were resolved by Day 7.

**Table 3:** Treatment-emergent Adverse Events by System Organ Class and Preferred Term

System organ class Preferred term, n (%)	NGX-4010 (n = 24)	Mild	Moderate	Severe
Number of subjects reporting one or more treatment-emergent AEs	24 (100.0)	6 (25.0)	14 (58.3)	4 (16.7)
General disorders and administration-site conditions	24 (100.0)	6 (25.0)	15 (62.5)	3 (12.5)
Application-site erythema*	24 (100.0)	9 (37.5)	15 (62.5)	0 (0)
Application-site pain*	22 (91.7)	4 (16.7)	15 (62.5)	3 (12.5)
Application-site edema*	4 (16.7)	3 (12.5)	1 (4.2)	0 (0)
Application-site pruritus*	1 (4.2)	1 (4.2)	0 (0)	0 (0)
Pain	1 (4.2)	0 (0)	0 (0)	1 (4.2)
Investigations	1 (4.2)	1 (4.2)	0 (0)	0 (0)
Blood pressure increased*	1 (4.2)	1 (4.2)	0 (0)	0 (0)
Musculoskeletal and connective tissue disorders	2 (8.3)	1 (4.2)	0 (0)	1 (4.2)
Back pain	2 (8.3)	1 (4.2)	0 (0)	1 (4.2)
Muscular weakness	1 (4.2)	1 (4.2)	0 (0)	0 (0)
Reproductive system and breast disorders	1 (4.2)	0 (0)	1 (4.2)	0 (0)
Breast mass	1 (4.2)	0 (0)	1 (4.2)	0 (0)
Skin and subcutaneous tissue disorders	3 (12.5)	3 (12.5)	0 (0)	0 (0)
Erythema	1 (4.2)	1 (4.2)	0 (0)	0 (0)
Rash papular	2 (8.3)	2 (8.3)	0 (0)	0 (0)

Note: counts indicate the numbers of subjects reporting one or more AEs that mapped to the MedDRA (version 10.1) system organ class. At each level of summarization, subjects were only counted once, according to the greatest severity. Abbreviations: AE; adverse event.

*AEs categorized as possibly or probably related to treatment.

**Figure 2:**
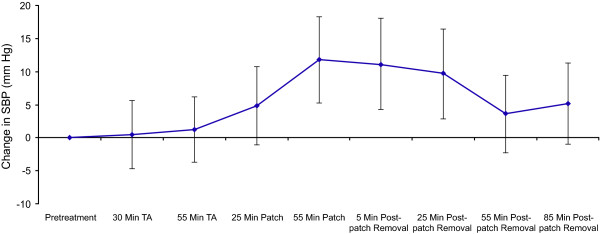
**Change in systolic blood pressure on the treatment day**. Blood pressure was recorded on the day of treatment before and during application of lidocaine 2.5%/prilocaine 2.5% cream, during NGX-4010 administration, and up to 85 minutes following patch removal. The results represent the change in mean (± 95% CI) systolic blood pressure from the mean pretreatment systolic blood pressure at each time point. CI; confidence interval, SBP; systolic blood pressure, TA; topical anesthetic.

No serious AEs or deaths occurred during the study period. A transient increase in mean blood pressure was observed during the application procedure (Figure 2). Blood pressure levels increased following administration of the NGX-4010 patch and reached the maximum mean increase in systolic blood pressure of 11.8 mm Hg, 55 minutes after application. Following patch removal, blood pressure levels gradually decreased toward the pretreatment level. At 85 minutes post-patch removal, the mean increase from pretreatment was 5.2 mm Hg. No changes in heart or respiratory rates were observed on the day of treatment.

#### Dermal Assessments

**Figure 3:**
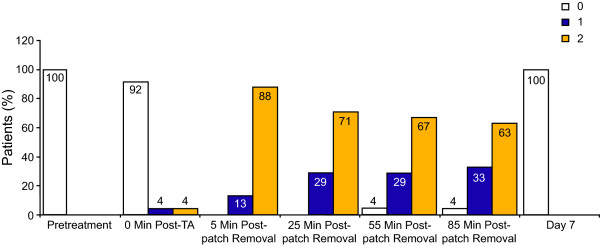
**Dermal irritation**. Dermal assessments were carried out to assess dermal irritation on the day of treatment before and immediately after application of lidocaine 2.5%/prilocaine 2.5% cream, at various time points following patch removal up to 85 minutes, and on study Day 7. Results show the percentage of patients at each time point with a dermal assessment score of 0 (no evidence of irritation), 1 (minimal erythema, barely perceptible), or 2 (definite erythema, readily visible; minimal edema, or minimal papular response), which was the maximum score recorded. Due to rounding, some figures do not add up to 100. TA; topical anesthetic.

Prior to application of the topical anesthetic, all patients had a dermal assessment score of 0, i.e. there was no evidence of erythema or dermal irritation (Figure 3). Following removal of the anesthetic cream, 2 patients had mild dermal irritation; 1 patient had a score of 1 (minimal erythema); and 1 patient had a score of 2 (definite erythema, minimal edema, or minimal papular response). Although all patients showed some dermal irritation after patch removal, no patients reached a score > 2. After patch removal, dermal irritation declined and there was no evidence of irritation in any patients on Day 7.

## Discussion

Based on assessment of the duration of NGX-4010 patch application, the pain experienced during treatment and the number of patients requiring analgesic medication to relieve treatment-related pain, the results of this study suggest that lidocaine 2.5%/prilocaine 2.5% cream is an acceptable pretreatment for 60-minute NGX-4010 applications. The mean duration of NGX-4010 application was 60.2 minutes and all patients received treatment for more than 90% of the intended treatment duration. This is comparable with previous trials in which patients were pretreated with 4% lidocaine cream where nearly all patients received treatment for 90% or more of the intended treatment duration [14,15,17].

In the present study, the pain experienced during and following NGX-4010 treatment was transient, with mean NPRS scores returning to pre-anesthetic levels (+0.7, 95% CI: -0.68, 2.09) within 85 minutes of patch removal. This result is similar to that of a previous report also showing a transient increase in pain during NGX-4010 application, and a return of mean NPRS scores to pre-anesthetic levels (+0.4) by 85 minutes post-patch removal [17]. The maximum mean increase in NPRS score of 3.0 (95% CI: 1.79, 4.21) observed just before removal of NGX-4010 in the present study was comparable with the mean NPRS score increases of 2.0 to 2.8 observed in a long-term safety study that included PHN patients [22]. Half of the patients used analgesic medication on the day of treatment for application site-related pain; this number rapidly decreased on subsequent days, with only 4 patients requiring medication on Day 1 and no patients requiring pain medication by Day 7. Nearly half of the patients (54%) used medication for treatment-related discomfort on Days 0-5. Again these results are similar to those previously observed in studies employing 4% lidocaine as pretreatment in patients with PHN, during which between 48 and 62% of patients used medication for treatment-related discomfort on Days 0-5 [14,15,17]. The level of dermal irritation that occurred during and after NGX-4010 application was also comparable with that which was observed in previous studies when 4% lidocaine was used as a pretreatment, where the majority of patients reported a maximum score of 2 or less [15,17].

The most common treatment-emergent AEs were application site-related pain and erythema. The incidence of application-site erythema in this study (100%) was similar to an incidence of 94% and 92% reported in previous studies of NGX-4010 in patients with PHN [14,17]. Application-site pain was reported by 92% of patients in the current investigation and was slightly higher than an incidence of 56% and 63% of patients reported in two previous studies [14,17]. However, despite this higher incidence of application-site pain, the pain increase and the use of medication for treatment-related discomfort was comparable, as described above. There was also a comparable effect on blood pressure during NGX-4010 application between patients pretreated with lidocaine 2.5%/prilocaine 2.5% cream and those in previous clinical trials pretreated with 4% lidocaine. Changes in blood pressure were associated with treatment-related changes in pain; in previous studies blood pressure increases were on average < 10 mm Hg [23], while in the current study the maximum mean increase was 11.8 mm Hg (95% CI: 5.3, 18.3).

The major limitation of this study was that it was an open-label, non-randomized trial that did not include a control arm or a comparator arm for the tolerability of NGX-4010 treatment following pretreatment with lidocaine 2.5%/prilocaine 2.5% cream. Therefore, direct comparisons between the tolerability of NGX-4010 treatment following pretreatment with lidocaine 2.5%/prilocaine 2.5% cream or 4% lidocaine cream cannot be drawn. This means that it is not possible to confirm whether NGX-4010 treatment is equally tolerable following pretreatment with either topical anesthetic cream. As there was no control arm, it is also not possible to compare directly the tolerability of NGX-4010 after lidocaine 2.5%/prilocaine 2.5% cream pretreatment with the tolerability after no anesthetic cream pretreatment. Consequently, it is not possible to determine whether pretreatment with lidocaine 2.5%/prilocaine 2.5% cream is essential for the tolerability of NGX-4010 treatment. However, the objective of this study was to investigate the tolerability of NGX-4010 treatment following pretreatment with lidocaine 2.5%/prilocaine 2.5% cream, because 4% lidocaine cream is not available in all of the countries in which NGX-4010 is approved. Despite the limitations of an open-label, non-randomized study, these objectives were met, with the data showing that NGX-4010 is a tolerable treatment following pretreatment with lidocaine 2.5%/prilocaine 2.5% cream. Other limitations of this study included the small sample size and lack of long-term follow-up. However, all application-site AEs in this study were short term and transient, resolving within 7 days and longer follow-up would not have provided additional tolerability information.

Based on the mode of action of lidocaine and prilocaine, it is not surprising to find that the tolerability of NGX-4010 following pretreatment with lidocaine 2.5%/prilocaine 2.5% appears to be similar to that seen when 4% lidocaine is used as a pretreatment. Both molecules are amide-type anesthetic agents, with similar chemical structures [24]. Both act on neuronal membranes by inhibiting sodium ion channels and consequently the ionic fluxes that are required for initiation and conduction of nerve impulses. Through inhibition of sodium ion influx, the threshold for nerve excitation is increased until the ability to generate an action potential is lost. Therefore, both lidocaine and prilocaine stabilize neuronal membranes of dermal pain receptors and nerve endings, resulting in the reduction of pain responses [24,25]. Previously, lidocaine 2.5%/prilocaine 2.5% cream has been shown to significantly reduce the burning pain of low-dose (0.075%) topically applied capsaicin [26], while axonal blockade with lidocaine can prevent the pain and hyperalgesia of intradermal capsaicin [27].

## Conclusions

In summary, NGX-4010 is a treatment option for PHN that may provide significant pain relief for up to 3 months from a single 60-minute application. Administration of the capsaicin 8% patch may be associated with application site-related pain that can be managed by pretreatment of the painful area with a topical anesthetic and the use of local cooling and short-acting oral analgesics. Previous clinical trials utilized 4% lidocaine cream as pretreatment; however, other topical anesthetics may also be used. Results from the current investigation provide evidence that NGX-4010 is tolerable following pretreatment with lidocaine 2.5%/prilocaine 2.5% cream. Moreover, tolerability appears comparable with the tolerability observed in the clinical trials using 4% lidocaine cream as pretreatment, in terms of intended application duration, extent of application site-related pain, use of medication for treatment-related discomfort, dermal irritation, AE profile, and transient blood pressure changes. Therefore, this study demonstrates that lidocaine 2.5%/prilocaine 2.5% cream is an acceptable alternative topical anesthetic for pretreatment prior to NGX-4010 application.

## Competing interests

Lynn R Webster is a consultant for NeurogesX Inc and Astellas Pharma Europe Ltd. Biao Lu, Jeffrey K Tobias and Geertrui F Vanhove are former employees of NeurogesX Inc and own NeurogesX Inc stock.

## Authors' contributions

LW, MN, MT and ED conducted the study, interpreted the data and edited the manuscript. BL, JT, and GV designed the study, analyzed and interpreted the data and prepared the manuscript. All authors read and approved the final manuscript.

## Pre-publication history

The pre-publication history for this paper can be accessed here:

http://www.biomedcentral.com/1471-2253/11/25/prepub
